# Health-related quality of life associated with daytime and nocturnal hypoglycaemic events: a time trade-off survey in five countries

**DOI:** 10.1186/1477-7525-11-90

**Published:** 2013-06-03

**Authors:** Marc Evans, Kamlesh Khunti, Muhammad Mamdani, Claus B Galbo-Jørgensen, Jens Gundgaard, Mette Bøgelund, Stewart Harris

**Affiliations:** 1 Department of Diabetes, University Hospital Llandough, Llandough Hospital, Penlan Road, Cardiff, Penarth, CF64 2XX, UK; 2 Diabetes Research Unit, University of Leicester, Leicester, LE1 7RH, UK; 3 Applied Health Research Centre (AHRC), Li Sha King Center and Department of Health Policy, Management, and Evaluation (Faculty of Medicine) and Leslie Dan Faculty of Pharmacy, University of Toronto, 30 Bond Street, Toronto, ON, M5B 1W8, Canada; 4 Incentive Partners, Holte Stationsvej 14, 1, Holte, DK-2840, Denmark; 5 Novo Nordisk A/S, Vandtårnsvej 114, Søborg, DK-2860, Denmark; 6 Schulich School of Medicine and Dentistry, Western University, 245-100 Collip Circle, UWO Research Park, London, ON, N6G 4X8, Canada

**Keywords:** Time trade-off, Hypoglycaemia, Quality of life, Disutility

## Abstract

**Background:**

Hypoglycaemic events, particularly nocturnal, affect health-related quality of life (HRQoL) via acute symptoms, altered behaviour and fear of future events. We examined the respective disutility associated with a single event of daytime, nocturnal, severe and non-severe hypoglycaemia.

**Methods:**

Representative samples were taken from Canada, Germany, Sweden, the United States and the United Kingdom. Individuals completed an internet-based questionnaire designed to quantify the HRQoL associated with different diabetes- and/or hypoglycaemia-related health states. HRQoL was measured on a utility scale: 1 (perfect health) to 0 (death) using the time trade-off method. Three populations were studied: 8286 respondents from the general population; 551 people with type 1 diabetes; and 1603 with type 2 diabetes. Respondents traded life expectancy for improved health states and evaluated the health states of well-controlled diabetes and diabetes with non-severe/severe and daytime/nocturnal hypoglycaemic events.

**Results:**

In the general population, non-severe nocturnal hypoglycaemic events were associated with a 0.007 disutility compared with 0.004 for non-severe daytime episodes, equivalent to a significant 63% increase in negative impact. Severe daytime and nocturnal events were associated with a 0.057 and a 0.062 disutility, respectively, which were not significantly different.

**Conclusions:**

This study applies an established health economic methodology to derive disutilities associated with hypoglycaemia stratified by onset time and severity using a large multinational population. It reveals substantial individual and cumulative detrimental effects of hypoglycaemic events – particularly nocturnal – on HRQoL, reinforcing the clinical imperative of avoiding hypoglycaemia.

## Background

Hypoglycaemia is defined as a deficiency of glucose in the bloodstream causing neuroglycopenic and autonomic symptoms, and is a well-recognized side effect in the insulin-based management of both type 1 and type 2 diabetes, with a reported overall incidence of 42.9 events per patient-year for type 1 diabetes and 16.4 events per patient-year for type 2 diabetes [1]. Biochemical definitions for low plasma glucose range between 3.0 mmol/L (European Medicines Agency) and 3.9–4.0 mmol/L (American Diabetes Association and Canadian Diabetes Association, respectively) [2-4]. Clinically, events can be subdivided into non-severe events, for which the individual is able to take remedial action, and severe events, for which third-party assistance is required. Episodes of hypoglycaemia may occur during the day (diurnally) or nocturnally, with the latter being particularly unpredictable. Hypoglycaemia symptoms often include, but are not limited to, trembling, hunger, sweating, difficulty concentrating, confusion, unconsciousness, or coma, and, in rare cases, death [5]. The debilitating effects of severe hypoglycaemia are widely acknowledged, but reports have also highlighted the negative impact of nocturnal events on patient wellbeing [6,7]. Hypoglycaemia can impact patient health-related quality of life (HRQoL) in two ways: firstly, through the direct physiological effects of repeated episodes; and secondly, through fear of future hypoglycaemia, leading to the adoption of avoidant, precautionary or compensatory actions (e.g., restricting holiday choices, avoiding physical exertion, defensive eating, insufficient insulin dosing) [7-10]. Moreover, repeated events can lead to impaired hypoglycaemia awareness, a condition affecting 20–25% of patients with type 1 diabetes and up to 10% of patients with type 2 diabetes, which raises the risk of severe hypoglycaemia and associated morbidity by up to six-fold [11].

To determine the HRQoL impact of hypoglycaemia, the impact per event must be quantified, which can be achieved by determining a ‘health utility’. Utilities enable HRQoL to be placed on a scale where a value of 1 corresponds to perfect health and 0 to death [12,13]. Although studies describing utility values for diabetes-related hypoglycaemic events exist, participant numbers have been generally small and the methods employed have suffered from various limitations; for example, a failure to estimate utility per event or to determine the impact of onset time or severity [14-18]. It is clear, therefore, that a significant knowledge gap remains regarding the relative utilities associated with a non-severe compared with a severe hypoglycaemic event, and how the timing of onset may further affect HRQoL.

The primary purpose of this study was to elicit a set of utility values for hypoglycaemic events using time trade-off (TTO) methods that can be used to assess the overall patient benefits of using appropriate diabetes medication. These utility values could be used directly as an input to diabetes decision models, and under certain assumptions can be used to estimate quality-adjusted life-years (QALYs) [19,20]. For example, in a guide to technology appraisal methods, the UK National Institute for Health and Clinical Excellence (NICE) advocates direct health-state valuations determined using the TTO method in a representative population sample in situations where EQ-5D™ data are unavailable [21].

The secondary objective was to examine the relative effects of diurnal and nocturnal hypoglycaemia on HRQoL, based on the hypothesis that nocturnal hypoglycaemic events, irrespective of severity, may have more impact than diurnal events due to their unpredictability.

## Methodology

### Time trade-off (TTO) methodology

Utility values are obtained by asking respondents to ‘trade off’ a portion of their remaining lifespan for an improved health state [19,22-29]. For example, respondents might choose between two options: A – to live in full health for 27 years and 0 months; or B – to have diabetes, experience non-severe daytime hypoglycaemia once a month, and live for 30 years and 0 months. If the respondent chooses option A, they are willing to exchange remaining lifetime (i.e., 3 years) to avoid living with diabetes and non-severe hypoglycaemia once a month, thereby indicating their utility in this health state is less than 0.9 (=27 years/30 years). Conversely, if they choose B, they indicate that their utility in this health state is more than 0.9. To make the trade-offs as realistic as possible, the time horizons used were based on each respondent’s projected life expectancy, obtained using the country, age and sex of the respondent at the time of the study, and the most recent World Health Organization life tables [30]. In order to test respondents’ understanding of the TTO concept, a test question was included offering a choice of full health and a longer remaining lifetime, or less than full health and a reduced lifetime. Respondents choosing the second option were excluded from analysis.

To identify the point of indifference (where both options are equally acceptable), respondents were asked the question repeatedly, varying only the number of years living in full health each time. This procedure followed standard bisection methodology, using a starting point of utility = 0.6 to reduce the utility value to an interval of 0.05.

Particular attention was given to the distribution tails. Respondents who either chose not to trade lifetime at a utility value of 0.95, or who were willing to trade a very high proportion of their remaining lifetime (0.875) to be restored to full health, were both carefully screened. Responses were excluded if the respondents refused to trade on ethical or religious grounds, or if they did not understand the question. However, those who believed the health state manageable or who stated a desire to live as long as possible due to obligations (e.g., caregivers) were retained.

### Definition of health states

The descriptions of hypoglycaemia health states were derived from a survey of 247 UK patients with diabetes. The survey was initially developed based on expert opinion garnered through an advisory board (which included all authors). The patients validated symptom descriptions for a non-severe daytime hypoglycaemic event, a non-severe nocturnal hypoglycaemic event and a severe hypoglycaemic event. In addition, they stated the level to which having hypoglycaemic events affected the frequency of certain diabetes-related and hypoglycaemia-related actions and worries in their daily lives. Together, this led to the definition of the 13 health states describing diabetes alone or diabetes combined with hypoglycaemia of differing event types and frequencies (Additional file 1: Table S1 and Additional file 2: Table S2).

### Survey description and methodology

Data were collected through an internet-based survey using an existing panel of prospective participants, an approach previously used successfully by other groups [31-33]. The panel covered a representative sample of the general populations in Canada, Germany, Sweden, the United States (US) and the UK. There remains a debate in the literature as to whether patient or public preferences carry the most influence when determining the value attached to a particular health state [34-36]. Therefore, to determine whether responses differed between the general population and people who may have personal experience of hypoglycaemia, a second population with type 1 diabetes and a third population with type 2 diabetes were also identified from the available panel. The study was carried out in accordance with the European Pharmaceutical Market Research Association code of conduct. Ethical approval was obtained from the University of Western Ontario Health Sciences Research Ethics Board in Canada (review number 181676) and Regionala Etikprövningsnåmnden, Lund, Sweden, where appropriate. All those contacted had previously agreed to participate in internet-based surveys. Various channels including web banners, telephone interviews and personal interviews were used for recruitment to ensure it remained representative. Only people aged 18 years or older were approached and took part at their discretion, and respondent anonymity was preserved throughout. Depending on the country, respondents were offered ‘points’ for online shopping or entry in a draw (nominal value 1–2 euros) as incentives to participate. The questionnaire was programmed in a commercial survey software package. To improve the answer quality and prevent unconsidered responses, a 10-second delay was introduced to pages containing a large amount of text. The survey was carried out in Swedish (Sweden), German (Germany), English (Canada, UK, and US) and French (Canada). Canadian respondents could choose between a French- or an English-language version. The questionnaire was translated into the respective language and back to English to ensure the veracity of each translation.

The functionality of the questionnaire was validated in a pilot study of 200 respondents. Following this, a few minor adjustments were made, including the addition of extra background questions and a question for screening respondents trading a very high proportion of their remaining life expectancy, and slight wording alterations to increase clarity.

Respondents were excluded based on the following criteria:

A. Those who failed a test question.

B. Inconsistency: those who valued the health state of baseline diabetes (i.e., without hypoglycaemia or other complications) worse than all three of the following health states:

•Diabetes and non-severe daytime hypoglycaemic events

•Diabetes and non-severe nocturnal hypoglycaemic events

•Diabetes and severe hypoglycaemic events.

In order to avoid respondent fatigue, individuals did not evaluate all 13 health states but were randomly assigned to:

•One health state of well-controlled diabetes

•One health state with non-severe daytime hypoglycaemia

•One health state with non-severe nocturnal hypoglycaemia

•One health state with severe hypoglycaemia (day or night).

In addition, respondents were required to provide their age and sex; to answer socioeconomic questions regarding their employment, household and region; and to complete an EQ-5D™ survey. Respondents with diabetes were asked to provide further information regarding the duration of diabetes, current medication, prevalence of hypoglycaemic events, awareness of hypoglycaemic events and current HbA_1c_ value or 7-day average blood sugar level. In the general population group, 1.8% and 8.7% of respondents had type 1 diabetes and type 2 diabetes, respectively.

### Statistical analysis

All statistical analyses were performed using SAS® version 9.2 statistical software. A utility value was assigned to each health state based on each individual response, derived from the midpoint of the indifference interval derived as described above. The average utility value was calculated for each health state, and a disutility per hypoglycaemic event derived by dividing the difference between the average utility and the baseline diabetes state utility by the number of annual events, to ensure that the resulting value reflected the effect of hypoglycaemia alone.

For each hypoglycaemic event class, up to four different event frequencies (health states) were evaluated (Additional file 2: Table S2). Unit values per hypoglycaemic incident type were calculated from the average TTO value for each frequency, weighted according to the distribution of those specific hypoglycaemic event frequencies among the participants with diabetes.

Statistical results for the type 1 and type 2 diabetes populations contain combined data from any general population respondents with type 1 or type 2 diabetes and data from the respective type 1 or type 2 diabetes populations.

As the response distribution was unknown but suspected to be non-normal, non-parametric bootstrapping was used to simulate standard errors and confidence intervals (CIs) for the mean TTO values. This method estimates the parameter’s distribution by repeatedly resampling the original data set with replacement [37-39]. For the present study, 10,000 iterations were performed.

Because it is rare for a patient with diabetes to experience severe hypoglycaemic events without non-severe events, some of the worries and limits to daily activities may already be accounted for by the disutility associated with non-severe events. Therefore, the initial disutility value (determined as the intercept in a regression model) for non-severe hypoglycaemic events was subtracted from the value obtained for severe hypoglycaemic events.

## Results

### Study population

Of the original 11,196 general population respondents who started the questionnaire, 10,087 (90%) completed it. A further 1178 respondents (10.5%) were excluded for failing the test question and 623 (6%) were excluded due to inconsistencies. The final general population sample was based on 8286 respondents, constituting 82% of the initial total, spread across five countries (1696 from Canada, 1607 from Germany, 1635 from Sweden, 1675 from the UK and 1673 from the US). The diabetes populations comprised 551 people with type 1 and 1603 with type 2. Table 1 summarizes background characteristics for each population.

**Table 1:** Respondent profile

**Characteristic**	**General population**	**Type 1 diabetes**	**Type 2 diabetes**
	**N (%)**	**N (%)**	**N (%)**
**Gender**			
Male	4237 (51)	308 (56)	898 (56)
Female	4049 (49)	243 (44)	705 (44)
**Mean age** (±SD)	46 ±16	39 ±14	54 ±12
**Mean EQ-5D™ score** (±SD)	0.81 ±0.25	0.72 ±0.30	0.70 ±0.31
**Occupation**			
In full-time education	550 (7)	58 (11)	44 (3)
Employed/self-employed	3912 (47)	243 (44)	516 (32)
Looking for paid work/government training scheme	444 (5)	10 (2)	28 (2)
Permanently unable to work due to long-term sickness/disability	595 (7)	29 (5)	72 (4)
Retired	1430 (17)	70 (13)	249 (16)
Looking after home or family	816 (10)	49 (9)	496 (31)
Other	539 (7)	47 (9)	135 (8)
**Household status**			
1 adult, no children	1761 (21)	126 (23)	373 (23)
1 adult, children	430 (5)	33 (6)	65 (4)
2 adults, no children	3060 (37)	166 (30)	729 (45)
2 adults, children	2042 (25)	152 (28)	263 (16)
3 adults, no children	482 (6)	31 (6)	81 (5)
3 adults, children	201 (2)	23 (4)	43 (3)
Other	310 (4)	20 (4)	49 (3)
**Total**	**8286 (100)**	**551 (100)**	**1603 (100)**

SD, standard deviation.

### Time trade-off results

For the baseline diabetes health state, 22% of the general population respondents (19–21% for the other health states) chose either not to trade or to trade all, placing them at the distribution extremes. Of these, 8% (8–11% for the other health states) were excluded from analysis according to the predefined criteria. Similar magnitudes of exclusion were seen in the diabetes populations.

The average TTO utility values for each health state are shown in Table 2 and Additional file 3: Figure S1. The utility value for well-controlled baseline diabetes was 0.844. Respondents considered living in a health state with severe or non-severe nocturnal hypoglycaemia worse than living in a health state with severe or non-severe daytime hypoglycaemia, irrespective of event frequency (Table 2). Similarly, they considered a health state with severe hypoglycaemia worse than a health state with an equivalent frequency of non-severe hypoglycaemia (for ‘one quarterly’, daytime, the values were 0.739 and 0.812 respectively).

**Table 2:** Average time trade-off utility values for health states [95% CIs, bootstrapped]

	**Frequency of hypoglycaemic events**					
**Health state**		**One a year**	**One a quarter**	**One a month**	**One a week**	**Three a week**
Baseline diabetes	0.844 [0.839 to 0.848]	–	–	–	–	–
Non-severe daytime hypoglycaemia	–	–	0.812 [0.802 to 0.822]	0.812 [0.802 to 0.822]	0.808 [0.799 to 0.817]	0.773 [0.762 to 0.784]
Non-severe nocturnal hypoglycaemia	–	–	0.809 [0.800 to 0.819]	0.804 [0.794 to 0.813]	0.775 [0.764 to 0.786]	0.729 [0.717 to 0.740]
Severe daytime hypoglycaemia	–	0.762 [0.751 to 0.773]	0.739 [0.739 to 0.750]	–	–	–
Severe nocturnal hypoglycaemia	–	0.759 [0.749 to 0.770]	0.738 [0.726 to 0.748]	–	–	–

Total for all countries, general population.

CI, confidence interval.

In general, as the frequency of hypoglycaemia increased, the utility value decreased, with the exception of non-severe daytime hypoglycaemia, where a frequency of one event quarterly resulted in the same utility as one event monthly (Table 2).

Table 3 shows the estimated average disutility associated with an annual hypoglycaemic event for the general population overall or stratified by country, and for the populations with either type 1 or type 2 diabetes.

**Table 3:** Disutility per hypoglycaemic event per year [95% CIs, bootstrapped]

	**Non-severe daytime event**	**Non-severe nocturnal event**	**Severe daytime event**	**Severe nocturnal event**
**General population**				
**Total**	0.004 [0.004 to 0.005]	0.007 [0.006 to 0.007]	0.057 [0.053 to 0.061]	0.062 [0.058 to 0.066]
**Canada**	0.006 [0.004 to 0.007]	0.008 [0.006 to 0.009]	0.059 [0.050 to 0.069]	0.062 [0.052 to 0.071]
**Germany**	0.002 [0.001 to 0.003]	0.004 [0.003 to 0.006]	0.060 [0.052 to 0.068]	0.066 [0.057 to 0.075]
**Sweden**	0.003 [0.001 to 0.004]	0.007 [0.005 to 0.008]	0.047 [0.038 to 0.055]	0.060 [0.052 to 0.069]
**UK**	0.005 [0.004 to 0.007]	0.008 [0.060 to 0.010]	0.062 [0.054 to 0.071]	0.066 [0.057 to 0.076]
**US**	0.005 [0.004 to 0.006]	0.007 [0.005 to 0.009]	0.055 [0.046 to 0.065]	0.057 [0.048 to 0.067]
**Populations with diabetes**				
**Type 1 diabetes**	0.004 [0.001 to 0.006]	0.008 [0.005 to 0.011]	0.047 [0.033 to 0.062]	0.051 [0.037 to 0.065]
**Type 2 diabetes**	0.005 [0.003 to 0.006]	0.007 [0.005 to 0.010]	0.060 [0.051 to 0.069]	0.078 [0.067 to 0.089]

CI, confidence interval.

**Figure 1:**
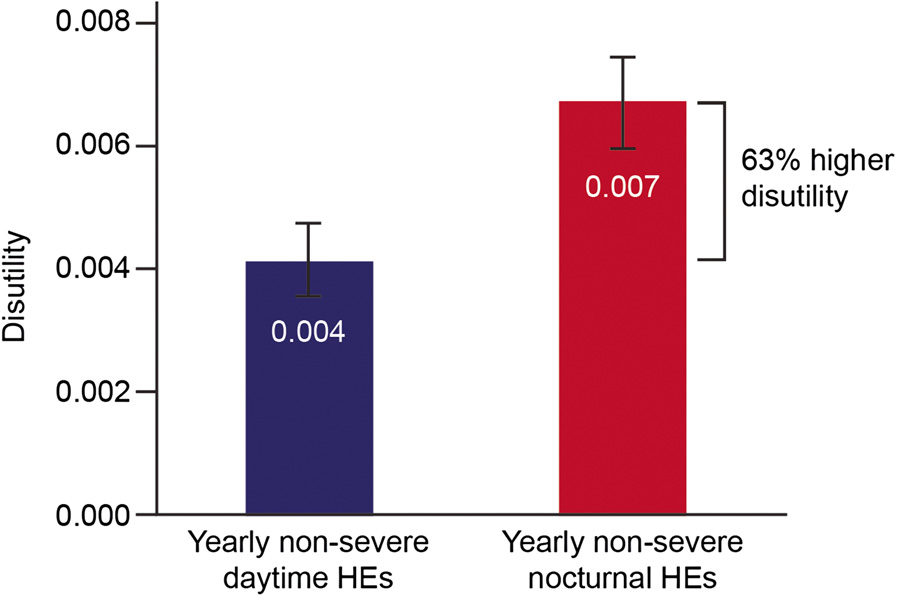
**Disutility associated with yearly incidence of non-severe daytime and nocturnal hypoglycaemic effects.**

Looking at the total population, a non-severe nocturnal hypoglycaemic event was associated with a disutility that was decreased by 0.003 (that is, from 0.007 to 0.004 [95% CI 0.002 to 0.003]) compared with non-severe daytime hypoglycaemia, equivalent to a significant 63.4% increase in negative impact (Figure 1). Severe nocturnal hypoglycaemia was associated with a disutility decrease of 0.006 (from 0.062 to 0.057 [95% CI −0.0001 to 0.0114]) compared with severe daytime hypoglycaemia, which was not statistically significant (Additional file 4: Figure S2).

When country-specific disutility values were compared with the overall values (Table 3), participants from Germany reported significantly lower disutilities (0.002 [95% CI 0.001 to 0.003] compared with 0.004 [95% CI 0.004 to 0.005] overall) for non-severe daytime hypoglycaemia (p = 0.00098) and non-severe nocturnal hypoglycaemia (0.004 [95% CI 0.003 to 0.006] compared with 0.007 [95% CI 0.006 to 0.007]; p = 0.0027). Similarly, Swedish participants also produced significantly lower disutilities for non-severe daytime hypoglycaemia (0.003 [95% CI 0.001 to 0.004] compared with 0.004 [95% CI 0.004 to 0.005] overall; p = 0.049) and severe daytime hypoglycaemia (0.047 [95% CI 0.038 to 0.055] compared with 0.057 [95% CI 0.053 to 0.061]; p = 0.032). In contrast, Canadian participants reported a significantly higher disutility for non-severe daytime hypoglycaemia (0.006 [95% CI 0.004 to 0.007] compared with 0.004 [95% CI 0.004 to 0.005] overall; p = 0.047). No country-specific significant differences in severe nocturnal hypoglycaemia disutilities were reported.

Overall, the disutilities obtained from the populations with type 1 or type 2 diabetes were similar to those of the general population. However, the type 2 diabetes population reported a significantly higher disutility for severe nocturnal hypoglycaemia (p = 0.008) compared with the general population (Additional file 5: Figure S3 and Additional file 6: Figure S4).

Correction of the initial disutility value for severe hypoglycaemic events resulted in an average reduction in the unit value for severe disutilities of 0.02 (ranging between 0.01 for Germany and 0.03 for UK; data not shown). However, this correction did not significantly affect the main findings.

## Discussion

The TTO method is a standard tool in the health economics evaluation arsenal, advocated for direct health-state valuations by health-technology assessment bodies. This study used a web-based TTO survey to determine the relative effects of severe, non-severe, daytime and nocturnal hypoglycaemic events on HRQoL and, as such, is the first analysis to provide a quantitative disutility value for each event class, and to demonstrate a clear increase in disutility for nocturnal compared with diurnal hypoglycaemic events.

As would be predicted based on clinical and patient-reported experiences, increasing event severity is associated with greater disutility. There were some variations between countries, with significantly lower disutilities reported for Germany and Sweden, and a significantly higher value reported for Canada for non-severe hypoglycaemia compared with the overall population. However, it is perhaps most interesting to note the high overall similarity between populations. The response consistency observed both across populations and between countries supports the credibility of the results and suggests that hypoglycaemia-related disutility is comparable and independent of healthcare system differences.

This study sampled a large number of respondents from multiple countries, 78–81% of whom were willing to trade-off health improvements against projected life expectancy, indicating understanding and acceptance of the TTO concept. The respondents represented three distinct groups: the general population, people with type 1 diabetes and people with type 2 diabetes, providing a unique opportunity to identify any significant differences. Indeed, a significantly higher disutility for severe nocturnal hypoglycaemia was reported by the type 2 diabetes population (p = 0.008).

The web-based approach, whilst facilitating respondent participation, does mean that help was not available if respondents had queries. Additionally, although a time delay was built into the survey, the lack of supervision may have led to some respondents not spending enough time considering their answers. Collectively, these design attributes may have led to inconsistencies within the responses. It should be noted, however, that the low dropout rate (10%) indicates the questionnaire was clear and manageable for most respondents, and potential skewing due to respondent fatigue was minimized by the methods employed.

Although participation bias must be considered, the incentives to participate would be expected to mitigate any initial disinclination. A recent report suggests that the application of discounting to correct for time preferences has an influential effect on outcomes [40]; we did not apply this in the present study. The use of an internet-based survey may also pose a selection bias, since only literate respondents with computer access could participate. However, the literacy rates and proportion of internet users in all sampled countries are high (e.g., the UK has a 99% literacy rate and 51.1 million computer users out of 63 million inhabitants) [41].

A further strength of this study is that the findings are of a similar magnitude compared with previous research. The observed baseline diabetes utility value of 0.844 found in the current study is in line with that reported previously [18]. A recent review found every non-severe event (day and night) may be associated with a utility loss of between 0.0033–0.0052 over 1 year [42]. However, previous studies describing diabetes-related utility values for hypoglycaemic events have various limitations. Cross-sectional studies using generic health instruments either suffer from potential unobserved confounding or do not estimate utility per event [14-16]. Matza and colleagues studied 129 people with type 2 diabetes, using the standard gamble technique, and found a significant disutility (p < 0.001) associated with the fear of hypoglycaemia overall, but did not distinguish between onset time or severity, or calculate disutility per event [17]. In contrast, Levy and colleagues surveyed 51 people with diabetes and 154 respondents without diabetes, using a TTO approach to estimate utility values per hypoglycaemic event, but did not report values for nocturnal or severe events [18]. In addition, these previous studies were limited by small sample sizes.

Moreover, the use of age-dependent, life-expectancy-based adaptation of the TTO questions for each respondent, applied successfully by other groups [32,33], may avoid some of the disadvantages of the artificial fixed 10- or 30-year horizon used elsewhere [18,22] by increasing the relevance of the trade-offs to the respondents and so providing more reliable utility value estimates.

Non-severe hypoglycaemic events have been shown to have a measurable detrimental impact on patient wellbeing, reflected by increased healthcare professional visits (25% of participants) and higher testing-strip consumption (5.6 on average), and a quarter of respondents reduced their insulin dose in the days immediately following an event [7]. After a non-severe nocturnal event, 23% of respondents reported arriving late or missing a day of work, and 32% missed a meeting or deadline [7]. Although an association between nocturnal hypoglycaemia and reduced HRQoL was demonstrated, the current investigation is the first analysis to provide quantitative disutility values and to demonstrate a clear increase in disutility for nocturnal compared with diurnal hypoglycaemic events.

Within clinical practice there is recognition of the phenomenon of ‘first being worst’; that is, the effect of each hypoglycaemic event on HRQoL diminishes as frequency increases and the patient adapts. In health economic terms, this is referred to as diminishing marginal disutility. Interestingly, the degree of disutility associated with increasing frequency of non-severe hypoglycaemic events consistently increased in this study, irrespective of onset time. The diminishing marginal disutility may reflect a coping mechanism, a maximum trade-off limit, or study design limitations, where some respondents might pay more attention to the health-state descriptions than the actual frequencies.

Identifying a minimally important difference (MID), described as the smallest change in the patient-reported outcome of interest that is either perceived as beneficial or that would elicit a change in behaviour [43,44], underpins the clinical relevance interpretation of any HRQoL study. However, no universally accepted method for MID estimation exists, with both anchor-based or distribution-based methods being employed [45-47]. An MID has been reported for some generic health instruments; Drummond reported an MID in utilities of 0.03 for the 15D instrument and the Health Utilities Index (HUI®), with the elaboration that utilities of 0.01 may be meaningful in some contexts [48]. Luo and colleagues reported mean MID estimates of 0.040 for the EQ-5D™ (US algorithm), 0.082 for the EQ-5D™ (UK algorithm), 0.045 for the HUI-2, 0.032 for the HUI-3 and 0.027 for the SF-6D [45]. Generic instruments generally lack sensitivity, which is why direct elicitation using an approach such as TTO becomes relevant. When patients trade a portion of their life expectancy to improve quality of life, they implicitly express the importance of the health state. In this study, the utility differences derived are per event; therefore, whilst they appear small initially, when the predicted annual event frequency is considered, the differences would be quite substantial.

## Conclusion

In summary, this analysis provides a unique evaluation of severe compared with non-severe hypoglycaemic events, and of the distinction between daytime and nocturnal hypoglycaemia, providing a comprehensive breakdown of the respective contributions made by each of these distinct event subgroups to patient HRQoL.

### Ethical approval

Ethical approval was granted by the University of Western Ontario Health Sciences Research Ethics Board in Canada (review number 181676) and Regionala Etikprövningsnåmnden, Lund, Sweden. No ethical approval was required for the other participating countries.

## Abbreviations

CI: Confidence interval; HRQoL: Health-related quality of life; MID: Minimally important difference; QALY: Quality-adjusted life-year; TTO: Time trade-off.

## Competing interests

The authors declare: financial support for the submitted work from Novo Nordisk A/S; ME has received consulting fees and travel support from Novo Nordisk and payment for lectures from Novo Nordisk, Bristol-Myers Squibb, Merck, Sharp & Dohme, sanofi-aventis, and Novartis; KK has received an NHS R&D study grant, served on advisory boards and received speaker’s fees from Novo Nordisk, Eli Lilly, Merck, Sharp & Dohme, Bristol-Myers Squibb, Astra Zeneca, Janssen, Boehringer, Takeda, Novartis, and Roche; MM does consultancy work for AstraZeneca, Bristol-Myers Squibb, Eli Lilly and Company, GlaxoSmithKline, Hoffman-La Roche, Novartis, Novo Nordisk, and Pfizer; CBGJ has received consulting fees including travel support and payment for reviewing the manuscript from Novo Nordisk and owns stock in Novo Nordisk; JG is employed by Novo Nordisk A/S and, as such, receives a salary and travel support for meetings related to the study; MB has received consulting fees including travel support and payment for reviewing the manuscript from Novo Nordisk; SH has received consulting fees, travel support, fees for advisory board membership, and speaker’s fees from Novo Nordisk, BMS/AZ, sanofi, Eli Lilly, Janssen, Takeda, Merck, and Novartis.

## Authors’ contributions

ME is the guarantor and takes responsibility for the integrity and accuracy of these data and the final decision to submit for publication. ME participated in the study design and interpretation, manuscript writing, and construction of tables and figures. KK, MM, and SH participated in the data interpretation and manuscript writing. CBGJ and MB participated in the study design; data collection, processing and analysis; manuscript writing, and construction of tables and figures. JG participated in the study design, analysis, interpretation, and manuscript writing. All authors read and approved the final manuscript.

## Supplementary Material

Additional file 1: Table S1 Health states: diabetes. Click here for file

Additional file 2: Table S2 Overview of the 12 hypoglycaemic health states (HHSs). Click here for file

Additional file 3: Figure S1 Visual representation of the utility estimates. Click here for file

Additional file 4: Figure S2 Disutility associated with yearly incidence of severe daytime and nocturnal hypoglycaemic events. Click here for file

Additional file 5: Figure S3 Disutility associated with yearly incidence of non-severe daytime and nocturnal hypoglycaemic events across the respective populations surveyed. Click here for file

Additional file 6: Figure S4 Disutility associated with yearly incidence of severe daytime and nocturnal hypoglycaemic effects across the respective populations surveyed. Click here for file
