# Supplementary material for: Health-related quality of life associated with daytime and nocturnal hypoglycaemic events: a time trade-off survey in five countries
Source: Health Qual Life Outcomes. 2013 Jun 3;11:90. doi: 10.1186/1477-7525-11-90 (PMC3679729; doi:10.1186/1477-7525-11-90)
Supplement: Additional file 1: Table S1 — Health states: diabetes. [file 1477-7525-11-90-S1.docx]

**Table S1. Health states: diabetes**

|  |  |  |
| --- | --- | --- |
| **Health state** | **Description:** | |
| Diabetes | Imagine that you have an illness called **diabetes**.   - This means that your body cannot keep your blood sugar at a constant level. - To control this, you often follow a special diet, are careful about eating regularly, and take steps to have something sugary nearby. - You take medication on a daily basis and sometimes need to plan your life around food and medicine. - You need to check your blood sugar from time to time. - You need to consider your diabetes when you are planning to exercise, travel, go out with friends, or drive. - Your diabetes does not affect your work/study, and you don't have any problems looking after yourself. - You occasionally worry about the effects of your diabetes on your day-to-day life.   [Patients with diabetes were also shown the following]   - Your diabetes is well controlled and you do not experience hypos (hypoglycaemic events) | |
