# Supplementary material for: Health-related quality of life associated with daytime and nocturnal hypoglycaemic events: a time trade-off survey in five countries
Source: Health Qual Life Outcomes. 2013 Jun 3;11:90. doi: 10.1186/1477-7525-11-90 (PMC3679729; doi:10.1186/1477-7525-11-90)
Supplement: Additional file 2: Table S2 — Overview of the 12 hypoglycaemic health states (HHSs). [file 1477-7525-11-90-S2.docx]

**Table S2. Overview of the 12 hypoglycaemic health states (HHSs)**

| **Health state** | **Description** | **Frequency and burden included** | **Frequency and burden included** | **Frequency and burden included** | **Frequency and burden included** |
| --- | --- | --- | --- | --- | --- |
| Non-severe nocturnal event | Imagine that besides having diabetes as described in the previous section you also experience so-called night-time hypoglycaemic events [frequency].  A night-time (nocturnal) hypoglycaemic event happens without warning during the night because of low blood sugar. You might wake up, feel shaky, hungry, irritable, extremely sweaty, confused, sick, and feel your heart pounding (have palpitations). This can be treated by eating or drinking something that contains sugar. You may have difficulty in getting back to sleep. You might have nightmares and when you wake up in the morning you might have a headache or be unusually tired for the whole of the following day.  Due to the fact that you are experiencing hypoglycaemic events [frequency]: | **HHS 1:**  once quarterly | **HHS 2:**  once monthly | **HHS 3:**  once weekly | **HHS 4:**  three-times weekly |
|  | – You [burden] plan your life around food and medication. | Often | Often | Almost always | Almost always |
|  | – You [burden] divert from your normal routine (medication and/or food intake) in order to avoid a hypoglycaemic event. This may cause negative effects on your health in the long run. | Occasionally | Occasionally | Fairly often | Fairly often |
|  | – You [burden] consider the risk of having a hypo when working, driving, exercising, traveling, and going out with friends. [Burden] you even limit these activities for fear of having a hypoglycaemic event. | Occasionally/  occasionally | Occasionally/  occasionally | Often/  sometimes | Often/  sometimes |
|  | - You [burden] ask others to check in on you during the night and worry about having to ask others to do so. | Occasionally | Occasionally | Fairly often | Fairly often |
|  | - You [burden] worry about the effects of your diabetes on your day-to-day life. | Sometimes | Sometimes | Almost always | Almost always |
| Non-severe daytime event | Imagine that besides having diabetes as described in the previous section you also experience so-called minor daytime hypoglycaemic events [frequency].  A minor daytime hypoglycaemic event happens suddenly because of low blood sugar. You might feel shaky, dizzy, sweaty, or irritable. You might also feel confused or sick, get a headache, or feel your heart pounding (have palpitations). These symptoms usually do not last long and will go away when you eat or drink something that contains sugar. After a minor daytime hypo, you might feel tired and not even remember what happened.  Due to the fact that you are experiencing minor daytime hypoglycaemic events [frequency]: | **HHS 5:**  once quarterly | **HHS 6:**  once monthly | **HHS 7:**  once weekly | **HHS 8:**  three-times weekly |
|  | – You [burden] plan your life around food and medication. | Often | Often | Almost always | Almost always |
|  | – You [burden] divert from your normal routine (medication and/or food intake) in order to avoid a hypo. This may cause negative effects on your health in the long run. | Occasionally | Occasionally | Fairly often | Fairly often |
|  | – You [burden] consider the risk of having a hypo when driving, working, exercising, traveling, and going out with friends. [Burden] you even limit these activities for fear of having a hypoglycaemic event. | Occasionally/ occasionally | Occasionally/ occasionally | Often/  sometimes | Often/  sometimes |
|  | – You [burden] ask others to check in on you during the day and worry about having to ask others to do so. | Rarely | Rarely | Sometimes | Sometimes |
|  | – You [burden] worry about the effects of your diabetes on your day-to-day life. | Occasionally | Occasionally | Often | Often |
| Severe events | Imagine that besides having diabetes as described in the previous section you also experience a so-called major hypoglycaemic event [frequency].  A major hypoglycaemic event happens suddenly because of very low blood sugar. You will not be able to treat it yourself; you will need help from another person, possibly medical assistance.  A major hypo may occur after a brief period of feeling shaky, dizzy, sweaty, irritable, or confused. You may experience seizures, convulsions, further confusion, fainting, or fall into a coma. In rare cases it is life-threatening.  Due to the fact that you are experiencing a major hypo [frequency]: | **HHS 9:**  once a year during the night | **HHS 10:**  once a year during the day | **HHS 11:**  quarterly during the night | **HHS 12:**  quarterly during the day |
|  | – You [burden] plan your life around food and medication. | Almost always | Almost always | Almost always | Almost always |
|  | – You [burden] divert from your normal routine (medication and/or food intake) in order to avoid a hypo. This may cause negative effects on your health in the long run. | Fairly often | Fairly often | Fairly often | Fairly often |
|  | – You [burden] consider the risk of having a hypo when driving, working, exercising, travelling, and going out with friends. [Burden] you even limit these activities for fear of having a hypo. | Often/  sometimes | Often/  sometimes | Often/  sometimes | Often/  sometimes |
|  | – You [burden] ask others to check in on you during the day and worry about having to ask others to do so. | Fairly often | Sometimes | Fairly often | Sometimes |
|  | – You [burden] worry about the effects of your diabetes on your day-to-day life. | Almost always | Often | Almost always | Often |
