# Supplementary figures and images for: Health-related quality of life associated with daytime and nocturnal hypoglycaemic events: a time trade-off survey in five countries
Source: Health Qual Life Outcomes. 2013 Jun 3;11:90. doi: 10.1186/1477-7525-11-90 (PMC3679729; doi:10.1186/1477-7525-11-90)

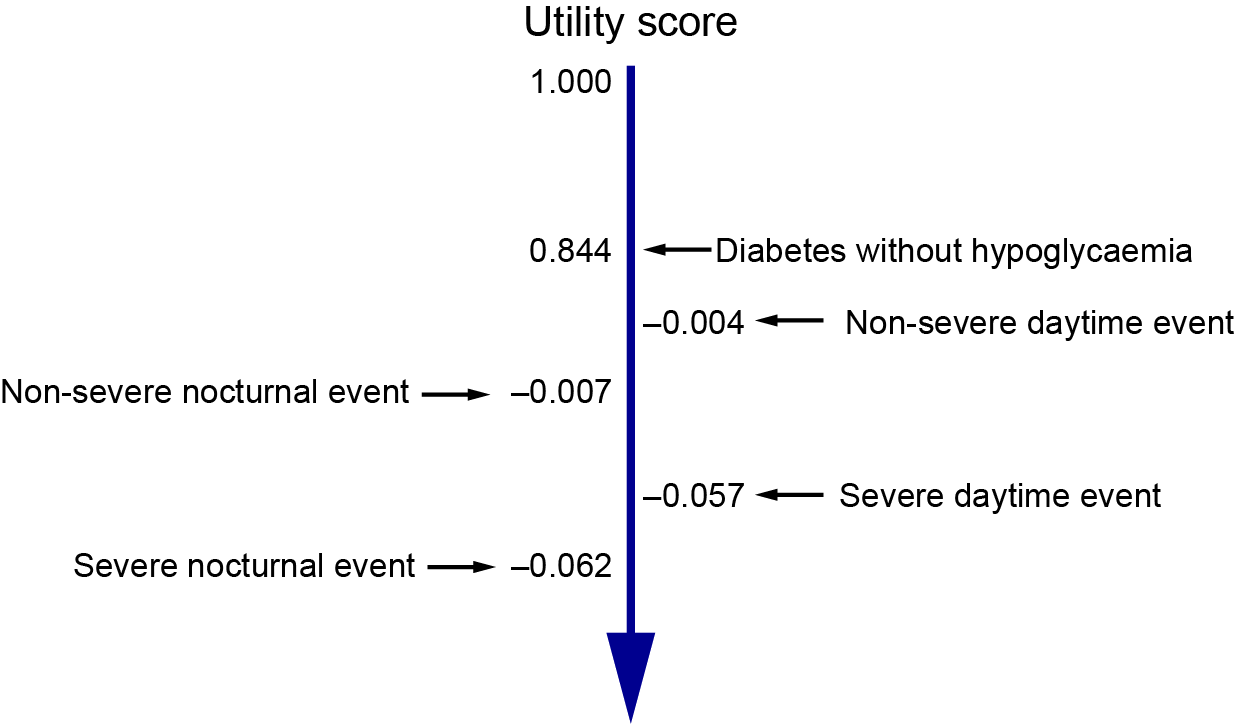


**Figure S1. Visual representation of the utility estimates.**

Supplement: Additional file 3: Figure S1 — Visual representation of the utility estimates. [file 1477-7525-11-90-S3.docx]
