# Supplementary material for: Health-related quality of life associated with daytime and nocturnal hypoglycaemic events: a time trade-off survey in five countries
Source: Health Qual Life Outcomes. 2013 Jun 3;11:90. doi: 10.1186/1477-7525-11-90 (PMC3679729; doi:10.1186/1477-7525-11-90)
Supplement: Additional file 4: Figure S2 — Disutility associated with yearly incidence of severe daytime and nocturnal hypoglycaemic events. [file 1477-7525-11-90-S4.docx]

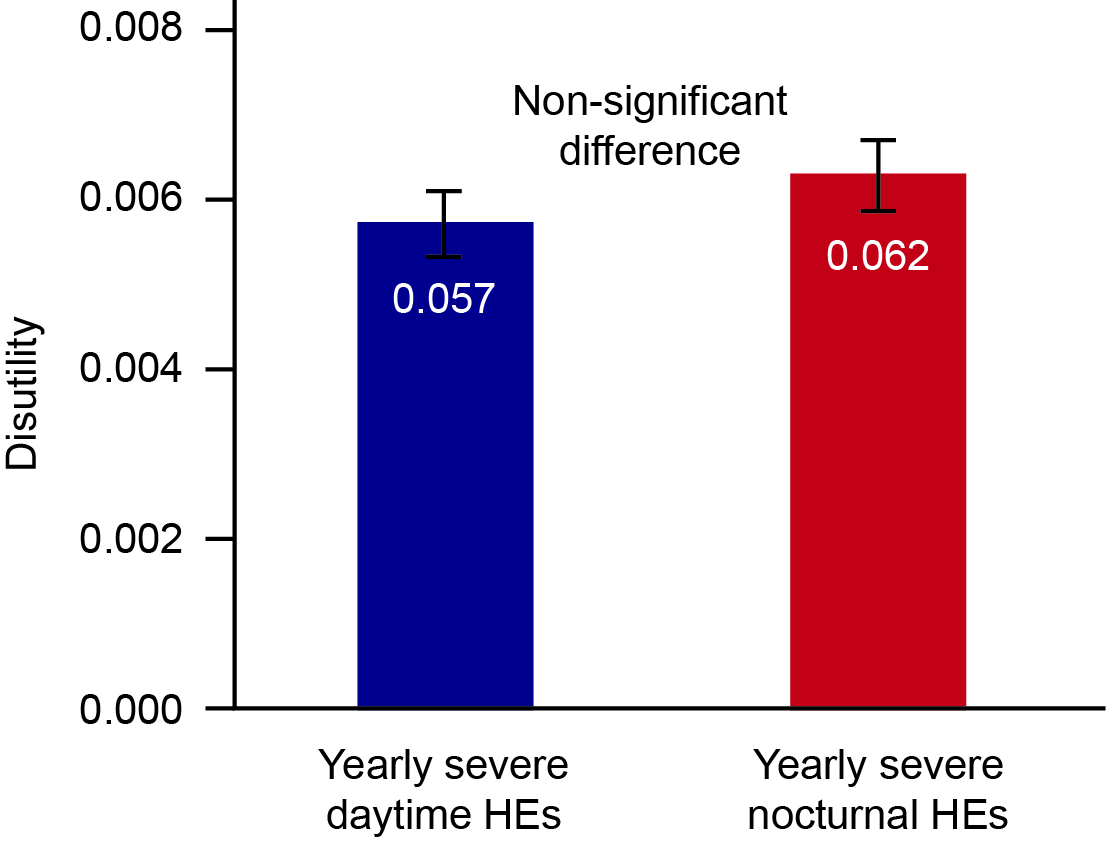


**Figure S2. Disutility associated with yearly incidence of severe daytime and nocturnal hypoglycaemic events.**

HE, hypoglycaemic event.
