# Supplementary material for: Health-related quality of life associated with daytime and nocturnal hypoglycaemic events: a time trade-off survey in five countries
Source: Health Qual Life Outcomes. 2013 Jun 3;11:90. doi: 10.1186/1477-7525-11-90 (PMC3679729; doi:10.1186/1477-7525-11-90)
Supplement: Additional file 5: Figure S3 — Disutility associated with yearly incidence of non-severe daytime and nocturnal hypoglycaemic events across the respective populations surveyed. [file 1477-7525-11-90-S5.docx]

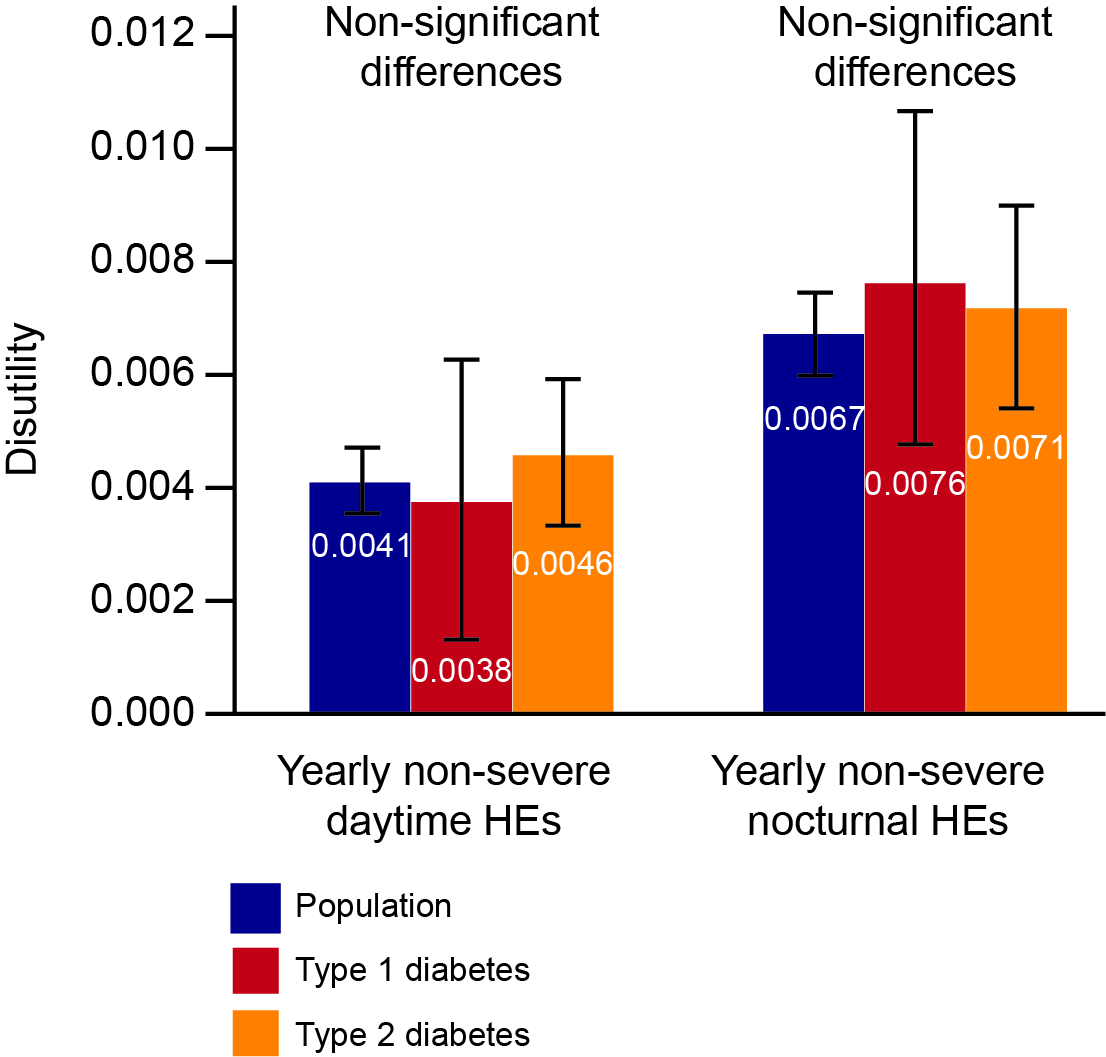


**Figure S3. Disutility associated with yearly incidence of non-severe daytime and nocturnal hypoglycaemic events across the respective populations surveyed**

HE, hypoglycaemic event
